# CpG DNA modulates interleukin 1β-induced interleukin-8 expression in human bronchial epithelial (16HBE14o-) cells

**DOI:** 10.1186/1465-9921-7-84

**Published:** 2006-06-01

**Authors:** N William Parilla, Valerie S Hughes, Kristin M Lierl, Hector R Wong, Kristen Page

**Affiliations:** 1 Division of Critical Care Medicine, Cincinnati Children's Hospital Medical Center, Cincinnati, OH, USA; 2 Department of Pediatrics, University of Cincinnati, Cincinnati OH, USA

## Abstract

**Background:**

Recognition of repeat unmethylated CpG motifs from bacterial DNA through Toll-like receptor (TLR-9) has been shown to induce interleukin (IL)-8 expression in immune cells. We sought to investigate the role of CpG oligodeoxynucleotides (ODN) on a human bronchial epithelial cells.

**Methods:**

RT-PCR and Western blot analysis were used to determine expression of TLR-9 in human bronchial epithelial cells (16HBE14o-). Cells were treated with CpG ODN in the presence or absence of IL-1β and IL-8 protein was determined using ELISA. In some cases cells were pretreated with chloroquine, an inhibitor of TLR-9 signaling, or SB202190, an inhibitor of the mitogen activated protein kinase p38, prior to treatment with IL-1β and CpG. TLR9 siRNA was used to silence TLR9 prior to treatment with IL-1β and CpG. IκBα and p38 were assessed by Western blot, and EMSA's were performed to determine NF-κB activation. To investigate IL-8 mRNA stability, cells were treated with IL-1β in the absence or presence of CpG for 2 h and actinomycin D was added to induce transcriptional arrest. Cells were harvested at 15 min intervals and Northern blot analysis was performed.

**Results:**

TLR-9 is expressed in 16HBE14o- cells. CpG synergistically increased IL-1β-induced IL-8 protein abundance, however treatment with CpG alone had no effect. CpC (a control ODN) had no effect on IL-1β-induced IL-8 levels. In addition, CpG synergistically upregulated TNFα-induced IL-8 expression. Silencing TLR9 using siRNA or pretreatment of cells with chloroquine had little effect on IL-1β-induced IL-8 levels, but abolished CpG-induced synergy. CpG ODN had no effect on NF-κB translocation or DNA binding in 16HBE14o- cells. Treatment with CpG increased phosphorylation of p38 and pretreatment with the p38 inhibitor SB202190 attenuated the synergistic increase in IL-8 protein levels. Analysis of the half-life of IL-8 mRNA revealed that IL-8 mRNA had a longer half-life following the co-treatment of CpG and IL-1β compared to treatment with IL-1β alone.

**Conclusion:**

Together, these data demonstrate that CpG modulates IL-8 synthesis in the presence of a pro-inflammatory mediator utilizing TLR9 and post-transcriptional mechanisms involving the activation of p38 and stabilization of IL-8 mRNA.

## Background

Cells of the immune system respond to bacterial cell components. These components termed pathogen associated molecular patterns (PAMPs) include lipopolysaccharide (LPS), peptidoglycan, lipotechoic acid, flagellin, bacterial lipoprotein, and DNA. Bacterial DNA has stimulatory sequences with a central cytosine-guanine (CG) core. Genomic DNA from bacteria, yeast and insects contain more unmethylated CG dinucleotides than mammalian genomic DNA. Unmethylated CG-rich DNA sequence motifs were developed and have been shown to stimulate mammalian immune cells [1-3]. Toll-like receptors (TLRs) are a family of receptors that function as pattern recognition receptors with the ability to recognize specific PAMPs. For example, TLR2 recognizes peptidoglycan, TLR3 recognizes double stranded RNA, TLR4 recognizes LPS, TLR5 recognizes flagellin, and TLR9 recognizes unmethylated DNA.

Cells within the innate immune (e.g. dendritic cells, macrophages, and B-lymphocytes) respond to CpG DNA [3]. CpG stimulation results in a signal transduction cascade involving activation of the nuclear factor (NF)-κB and activator protein (AP)-1 pathways [4,5]. However, not all cells of the innate immune system respond uniformly to CpG DNA stimulation. For example, CpG DNA strongly activates extracellular regulated kinase (ERK) 1/2 in macrophages, whereas CpG DNA causes a marginal activation of ERK 1/2 in dendritic cells [6]. In murine B lymphoma cells, CpG induces activation of c-Jun NH2-terminal kinase (JNK) and p38, but not ERK [5]. There are a few reports examining epithelial cell signaling responses to CpG. In a human colon-derived crypt-like HT-29 cell line, *E. coli*-derived DNA induced AP-1 translocation which involved Fos [7]. In the respiratory cell line 1HAEo-, *E.coli*-derived DNA induced a 2-fold increase in NF-κB translocation as determined by luciferase assay [8]. A variety of CpG sequences have been used in the abovementioned studies which could lead to differences in signaling.

The epithelium is a barrier to the entry of pathogens, and as a dynamic system for host response, the epithelium can produce natural antimicrobial factors and release pro-inflammatory cytokines. Therefore it is thought that the airway epithelium plays a role in modulating innate immunity. There have been a few studies to date that have investigated the role of CpG on regulating cytokine release in airway epithelial cells. For the most part, the responses have been small, i.e. CpG induces roughly a 2-fold increase in IL-8 production in human respiratory epithelial cells (1HAEo-) and a tracheal epithelial cell line derived from a patient with cystic fibrosis (CFTE29o-) [8,9]. In BEAS-2B cells, TLR9 was among the least highly expressed TLRs, and CpG had no effect on regulating cytokine production [10].

In the setting of acute (pneumonia) and chronic (cystic fibrosis) lung disease, bacteria and bacterial components (including DNA) comprise the pulmonary milieu; moreover, pro-inflammatory mediators are also likely to be present. Physiologically, the body would not encounter bacterial DNA alone, it would be in the presence of organisms and inflammatory mediators. Therefore we were interested in the role of CpG in the presence of other pro-inflammatory molecules. Since interleukin IL-1β is one of a family of cytokines involved in a variety of acute and chronic diseases, we hypothesized that CpG DNA would potentiate the IL-1β response in bronchial epithelial cells. In this study, we evaluated the effect of CpG DNA on IL-1β-induced IL-8 expression in SV40- transformed human bronchial epithelial cells (16HBE14o-) cells.

## Materials and methods

### Cell culture

SV40-transformed human bronchial epithelial cells (16HBE14o-) were grown as previously described [11]. Cells were treated with human IL-1β (Roche Applied Science, Indianapolis, IN), human TNFα (R&D, Minneapolis MN), synthetic CpG (5' TCG TCG TTC CCC CCC CCC CC 3'), or CpC (5' TCC TCC TTC CCC CCC CCC CC 3' ODN (TriLink, San Diego, CA) with a phosphodiesterase backbone. The CpG sequence is the 2080 CpG sequence identified by Hartmann and Krieg to be a potent activator of human B cells [12].

### RT-PCR

RNA from untreated growing cells was extracted with TRIzol and RT-PCR was performed [13]. TLR9 primers used were sense 5'-AAGGCCAGGTAA TTGTCACG-3'and antisense 5'-AGCAGCTCTGCAGTACGTC-3' (PCR product, 224 bp) [14]. PCR amplification for all primers was performed for 40 cycles of 94°C for 15 sec, 58°C for 30 sec, 72°C for 45 sec, followed by one cycle of 72°C for 10 min. Product was run on a 1.5% agarose gel containing 10 μg ethidium bromide.

### ELISA

Cells were treated with CpG (0.3 μM – 3 μM), control CpC (3 μM), IL-1β (0.1 ng/ml), or TNFα (3 ng/ml) either alone or in combination for 16 h. In separate experiments, cells were pretreated with chloroquine (**1**-10 μg/ml; Sigma, St. Louis, MO) or SB202190 (0.3–3 μM; Calbiochem, La Jolla, CA) 1 h prior to treatment. In some experiments, cells were treated with poly (I:C) (1–10 μg/ml from Roche Applied Science, Indianapolis, IN) or lipopolysaccharide (LPS; 0.1 – 10 μg/ml from Sigma, St. Louis, MO) in the absence or presence of IL-1β. Cell supernatants were collected and clarified (13,000 rpm for 10 min at 4°C) prior to being analyzed for IL-8 by ELISA (Biosource, Camarillo, CA).

### Immunoblot analysis

Growing cells were washed in PBS and extracted in a lysis buffer as previously described [13]. Extracts (30 μg) were resolved on a 10% SDS-polyacrylamide gel and transferred to nitrocellulose. After incubation with primary antibody (TLR9, Santa Cruz, Santa Cruz, CA; phospho-p38, Biosource, Camarillo, CA; p38, Cell Signaling Technology, Beverly, MA) signals were amplified and visualized using enhanced chemiluminescence.

### Transfection of siRNA

Cells were transfected using Lipofectamine 2000 (Invitrogen, Carlsbad, CA) and either a negative control siRNA (Qiagen, Valencia, CA) or siRNA for TLR9 (Ambion, Austin TX; three TLR9 siRNA's were purchased and mixed together). Cells were transfected at 30–50% confluence in Optimem according to the protocol from Invitrogen. Seventy two hours following transfection, TLR9 mRNA levels were determined by RT-PCR. For the ELISA, 48 h following tranfection, cells were deprived of serum for 8 h and then treated with CpG (3 μM) in the absence or presence of IL-1β (0.1 ng/ml) for 16 h.

### Electrophoretic mobility shift assay (EMSA)

Cells were treated with CpG (0.3 μM) and/or IL-1β (0.1 ng/ml) for 1 h. Nuclear extraction procedures were performed as described [15]. 4 μg of nuclear proteins were preincubated with binding buffer and 100,000 counts/min of [γ^32^P]-NF-κB probe (Santa Cruz), and incubated on ice for 15 min. Five-fold cold NF-κB probe was added for specific competition, and AP-1 was added for non-specific competition. Nuclear extracts were added and incubated at RT for 15 min. Protein-nucleic acid complexes were resolved, transferred to nitrocellulose, dried and exposed to photographic film.

### IL-8 mRNA stability

Cells were treated with IL-1β (0.1 ng/ml) in the absence or presence of CpG (0.3 μM) for 2 h at which time media was changed and actinomycin D (2 μg/ml) was added to the cells to induce transcriptional arrest. One dish of cells were harvested for total RNA isolation at 15 min intervals.

### Northern blot analysis

Total RNA was isolated using TRIzoll reagent (Gibco-BRL, Rockville, MA), and RNA (15 μg) was separated on a 1% agarose/3% formaldehyde gel, transferred to nylon membranes, and ultraviolet auto-cross-linked (UV Stratalinker 1800) as previously described [16]. Membranes were prehybridized for 4 h at 42°C and subsequently hybridized overnight with a radiolabeled IL-8 cDNA probe [17]. The cDNA probe was labeled with [α-^32^P]dCTP (specific activity 3,000 Ci/mM, NEN Research Products) by random priming. Membranes were subsequently washed twice with saline-sodium citrate-0.1% SDS at 53°C and developed.

### Statistical analysis

Statistical significance was assessed by one-way analysis of variance (ANOVA) and differences were pinpointed by Student-Newman-Keuls' multiple range test.

## Results

### TLR9 is expressed in human bronchial epithelial cells

**Figure 1:**
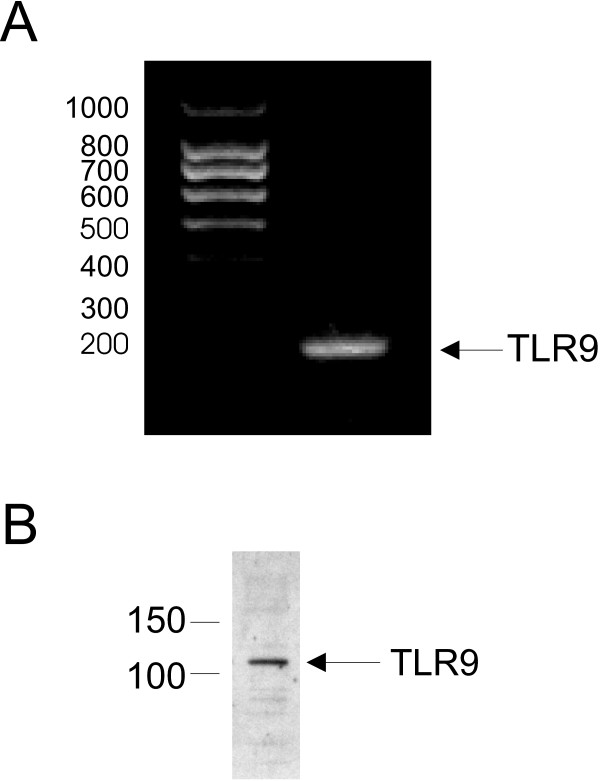
TLR9 expression in human bronchial epithelium. A. Total RNA from 16HBE14o- was extracted, reverse-transcribed, and amplified with specific primers for TLR9 and β-actin. B. Whole cells were extracted and western blot analysis was performed using an antibody against TLR9. Each experiment was performed twice and a representative experiment is shown.

We confirmed a previous report showing that the human airway epithelial cell line (16HBE14o-) expresses TLR-9 by performing RT-PCR for mRNA and Western blot for protein [9]. TLR9 mRNA was constitutively expressed (Figure 1A) and TLR9 protein was detected (Figure 1B).

### CpG augments IL-1β-mediated IL-8 production

**Figure 2:**
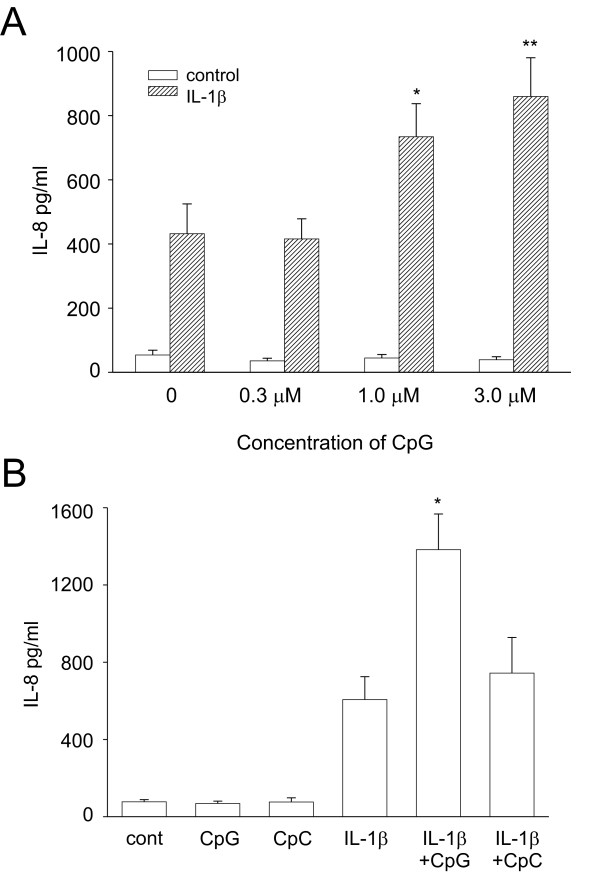
CpG synergistically increased IL-1β-induced expression of IL-8. A. 16HBE14o- cells were treated with increasing concentrations of CpG in the absence or presence of IL-1β (0.1 ng/ml). Cell supernatants were harvested and analyzed for IL-8 by ELISA. Data represent means ± SEM for 6 separate experiments (compared to IL-1β alone, *p = 0.001, **p = 0.008, ANOVA). B. Cells were treated with CpG or control CpC in the absence or presence of IL-1β. Data represent means ± SEM for 5 separate experiments (compared to IL-1β alone, *p = 0.038, ANOVA).

We hypothesized that exposure to CpG in the presence of IL-1β may have pro-inflammatory effects on human airway epithelium. We treated cells with increasing doses of CpG in the absence or presence of IL-1β. Treatment of cells with ODNs alone had no effect on IL-8 release as determined by ELISA. However, in the presence of IL-1β, CpG synergistically increased IL-8 release (Figure 2A). A dose-dependent increase was detected using CpG concentrations of 0.3 to 3 μM; however, we chose 3 μM for the rest of our studies. A range of IL-1β concentrations from 10 pg/ml to 10 ng/ml were originally tested. The concentration of 0.1 ng/ml IL-1β was chosen since that dose gave a submaximal increase in IL-8 expression (data not shown) and showed the largest level of synergy (data not shown). To confirm the specificity of the CpG, control ODN (CpC) was used. CpC had no effect on IL-1β-induced release of IL-8 (Figure 2B). Endotoxin was not detected in the synthetic ODN preparations (data not shown).

To investigate the specificity of this synergy, we also treated cells with poly (I:C) which binds TLR3, or LPS which binds TLR4 in the absence or presence of IL-1β. Either alone, or in the presence of IL-1β, poly (I:C) had no effect on IL-8 production (data not shown). LPS had minimal effects on IL-8 production and did not induce synergy when co-treated with IL-1β (data not shown).

### CpG synergistically increased TNFα-induced IL-8 expression

**Figure 3:**
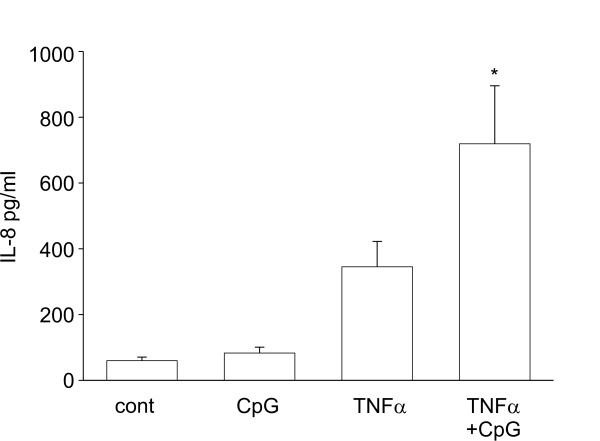
CpG synergistically increased TNFα-induced expression of IL-8. Cells were treated with CpG in the absence or presence of TNFα. Cell supernatants were harvested and analyzed for IL-8 by ELISA. Data represent means ± SEM for 4 separate experiments (compared to TNFα alone, *p = 0.026, ANOVA).

Because bacterial DNA would exist in the presence of a variety of pro-inflammatory mediators, we hypothesized that CpG would also enhance the signaling of other cytokine mediators. As predicted, CpG augmented TNFα-induced IL-8 expression similar to the effect of IL-1β (Figure 3).

### TLR9 inhibition attenuated CpG induced IL-8 expression

**Figure 4:**
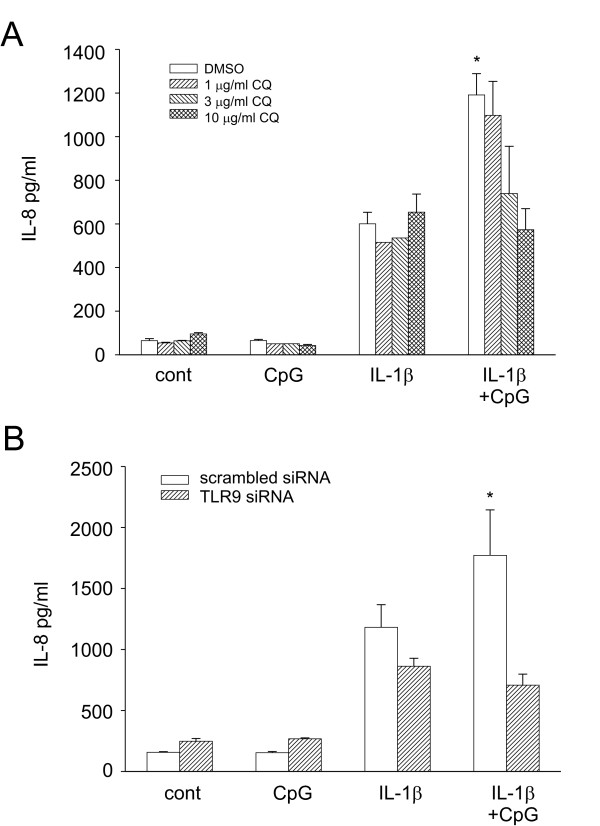
Inhibition of TLR9 attenuated CpG-induced synergy. A. Cells were treated with increasingconcentrations ofchloroquine (CQ; **1**-10 μg/ml) for 1 hr prior to addition of CpG and/or IL-1β. Data represent means ± SEM for 3–7 separate experiments (compared to IL-1β alone, *p = 0.012, ANOVA). B**. **Cells were transfected with scrambled siRNA or TLR9 siRNA. 48 h later, cells were depleted of serum for 8 h and then treated with IL-1β and/or CpG for 16 h. Cell supernatants were harvested and analyzed for IL-8 by ELISA. Data represent means ± SEM for 4 separate experiments (compared to IL-1β alone, *p = 0.003, ANOVA). There was no statistical difference between IL-1β treatment in the scrambled siRNA vs TRL9 siRNA.

Chloroquine, an inhibitor of endosomal acidification, has been shown to inhibit TLR9 signaling [7]. To confirm that CpG signals through TLR9, we pre-treated cells with increasing concentrations of chloroquine one hour before treatment with IL-1β, CpG or both. Chloroquine had no effect on IL-1β induced IL-8 expression. However, inhibition of TLR9 signaling abolished CpG's synergistic effect (Figure 4A). To further confirm the role of TLR9, we used siRNA to selectively silence TLR9. TLR9 siRNA resulted in greater than a 90% decrease in TLR9 mRNA compared to scrambled siRNA (data not shown). Importantly, silencing TLR9 using siRNA resulted in the loss of CpG-induced synergy of IL-8 (Figure 4B). Together, these data implicate TLR9 mediating signaling in CpG-induced synergy.

### CpG does not activate NF-κB

**Figure 5:**
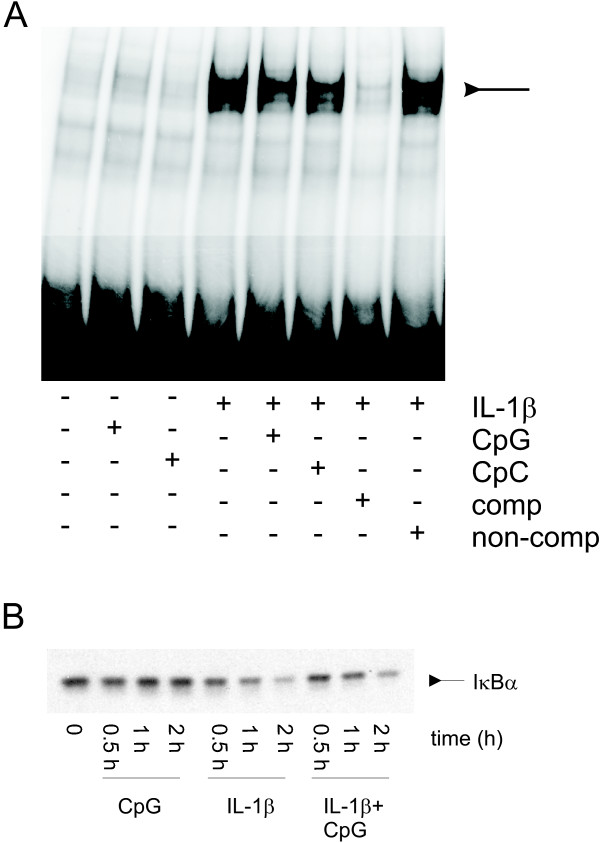
CpG does not induce NF-κB translocation or DNA binding. A. Cells were treated with CpG, CpC, and/or IL-1β for 1 hr. Nuclear extracts were obtained, incubated with a ^32^P end-labeled double-stranded NF-κB oligonucleotide probe, and resolved on a gel. Unlabed NF-kB probe (comp) or AP-1 probe (non-comp) was added at 5 times the concentration of labeled probe to show specificity. This experiment was repeated twice. B. Cells were treated with CpG and/or IL-1β for 0.5, 1, or 2 h prior to extraction and resolution on a gel. Immunoblot analysis using an antibody against IκBα is shown. This experiment was repeated four times.

We next asked whether CpG increased NF-κB translocation to the nucleus. In these experiments, we directly examined the effect of CpG on IL-1β-mediated nuclear translocation of NF-κB. Treatment with IL-1β increased nuclear translocation of NF-κB as determined by EMSA, however CpG alone had no effect. Concomitant treatment with CpG and IL-1β did not alter NF-κB nuclear translocation compared with that in cells treated with IL-1β alone (Figure 5A). We next measured the degradation of IκBα. Treatment with IL-1β caused degradation of IκBα compared to control cells. CpG alone did not alter IκBα degradation. Concomitant treatment with CpG and IL-1β did not alter IκBα degradation or the time of reappearance of IκBα (Figure 5B). There was no statistical difference between the IκBα protein levels when comparing IL-1β and IL-1β plus CpG treatment (data not shown). In addition, control CpC had no effect alone or in combination with IL-1β (data not shown).

### CpG increased p38 MAPK activation

**Figure 6:**
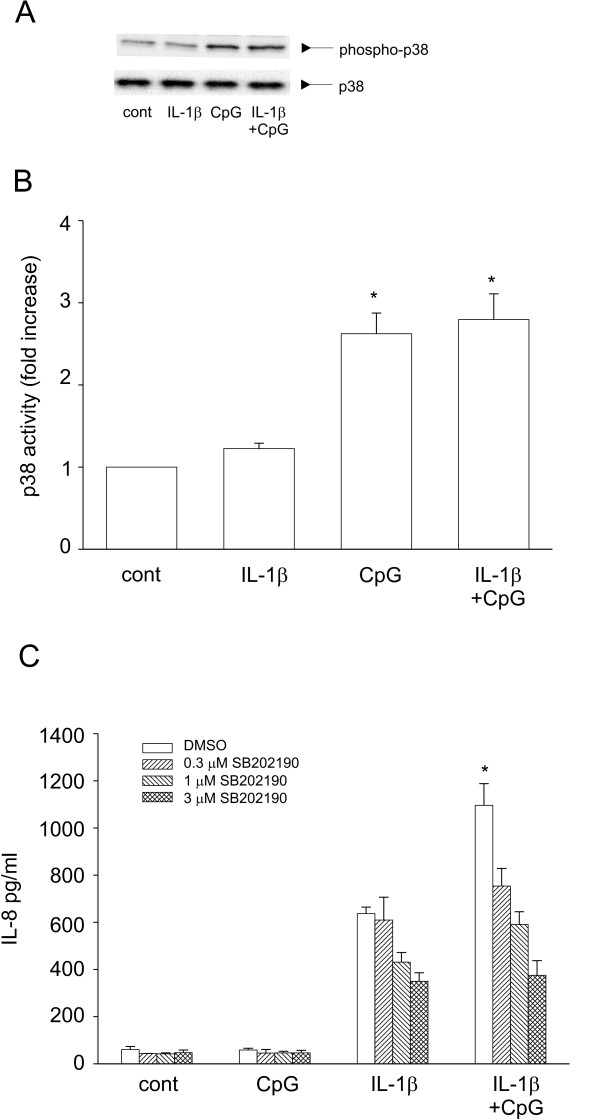
CpG-induced IL-8 is dependent on activation of p38. A. Cells were treated with IL-1β, CpG alone or in combination for 1 h and Western blots were performed for phospho-p38 (top blot) and total p38 (bottom blot). A representative experiment is shown. B. Normalization of five separate experiments (means ± SEM). Compared to control, *p < 0.001; CpG compared to CpG plus IL-1β is not statistically different p = 0.555). C. Cells were pretreated with increasing concentrations ofSB202190 (0.3-3 μM for 1 h) prior to addition of IL-1β or CpG. Cell supernatants were harvested and analyzed for IL-8 by ELISA. Data represent means ± SEM for 3–7 separate experiments (compared to IL-1β alone, *p > 0.001, ANOVA).

We next tested the role of CpG in activating p38, a signaling intermediate known to increase the stability of IL-8 mRNA levels. Cells treated with CpG show an increase in p38 phosphorylation, as determined by Western blot analysis. Alone, IL-1β had little effect on p38 phosphorylation. Co-treatment of IL-1β with CpG did not further augment p38 phosphorylation (Figure 6A and 6B). Pretreatment of cells with SB202190, a p38 inhibitor, reduced IL-1β-induced IL-8 expression. Importantly, SB202190 totally abolished CpG-induced synergy (Figure 6C). To test if CpG had an effect on ERK activation, we measured ERK phosphorylation following CpG treatment in the presence or absence of IL-1β. There were no significant changes in ERK phosphorylation following CpG treatment (data not shown).

### CpG increases the stability of IL-8 mRNA levels

**Figure 7:**
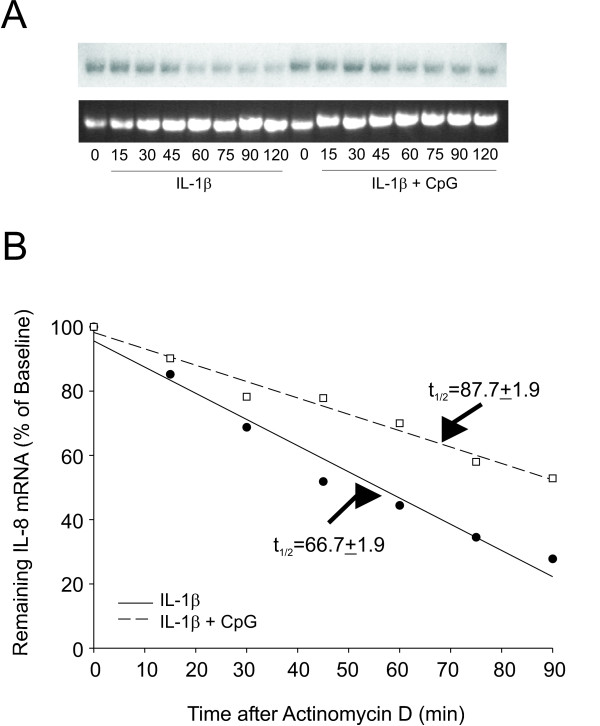
CpG increased IL-1β-induced IL-8 mRNA stability. A. Cells were treated with IL-1β in the absence or presence of CpG for 2 h at which time media was changed and actinomycin D was added to the cells to induce transcriptional arrest. Both groups of cells were harvested for total RNA extraction at 15 min intervals, as indicated. The top gel is a representative Northern blot and the bottom gel shows the 18s rRNA levels depicted by ethidium bromide staining of the transferred membrane. B. Decay rate of Northern blot shown in A. Data points are plotted as the percent IL-8 mRNA remaining relative to time zero. Half-life calculations are depicted as the average (± SEM, n = 4 separate experiments) time at which there was 50% IL-8 mRNA remaining under the respective treatments.

We next tested if CpG influenced the stability of IL-8 mRNA. For this, we treated cells with IL-1β in the absence or presence of CpG for 2 h. Actinomycin D was added to inhibit further transcription, and cells were incubated to allow for mRNA decay. Total RNA was isolated in 15 min increments for a period of 2 h. The half-life of IL-8 mRNA following treatment with IL-1β alone was 66.7 ± 1.9 min. When CpG was added with IL-1β, the half life of IL-8 mRNA increased to 87.7 ± 1.9 min (Figure 7A and 7B).

## Discussion

Bacterial DNA has been shown to have immunostimulatory effects on cells of the innate immune system, in particular dendritic cells, macrophages, and B-lymphocytes [3]. There have been a few reports showing that CpG or *E. coli *DNA alone induced IL-8 production in airway epithelial cells [8,9]. However these effects were minimal (roughly 2-fold) compared to other activators such as TNFα which increased IL-8 production by 10–20 fold in 16HBE14o- cells [18,19]. In BEAS-2B cells, treatment with CpG had no effect on regulating cytokine production (10) similar to our findings. It is conceivable that cells would not be exposed to just bacterial DNA without other mediators present (i.e. LPS from the bacteria, or pro-inflammatory mediators from the host defense system). The present study shows that CpG, while having no effect alone, augments IL-1β- and TNFα-induced IL-8 production in human bronchial epithelial cells. In this report, we used a CpG (2080) which is a 20-mer phosphodiester ODN with a thymidine located between the two CpG dinucleotides [12]. Hartmann has previously shown that this motif resulted in a slightly higher activity than having an adenine between the two CpG motifs [12]. CpG ODN sequences with a phosphodiester backbone have been shown to bind TLR9 more specifically than ones having a phosphorothioate protected backbone [20,21].

To confirm the role of TLR9 signaling in CpG-mediated regulation of IL-8, we transfected cells with siRNA against TLR9. This resulted in greater than 90% decrease in TLR9 mRNA levels. When TLR9 was silenced, CpG treatment no longer resulted in the synergistic activation of IL-1β-induced IL-8 protein release. These data suggest the importance of TLR9 in this effect. In addition, activation of TLR3 or TLR4 in the presence of IL-1β had no further effect on IL-8 production.

IL-8 is regulated transcriptionally by NF-κB, AP-1 and NF-IL6. There is an absolute requirement for NF-κB in IL-8 promoter regulation. In immune cells, CpG increased NF-κB activation and IL-8 synthesis [4,5]. In previous studies performed using airway epithelial cells, one group reported that CpG did not increase NF-κB reporter gene expression [9], while another group reported that *E.coli *DNA increased NF-κB reporter gene expression by almost 2-fold using 100 μg/ml *E. Coli *DNA [8]. In the present study we used 18.5 μg/ml (0.3 μM, a concentration chosen based on its level of synergy with IL-1β) CpG and we did not detect an increase in NF-κB, however we did not test higher concentrations nor did we perform any experiments with *E. coli *DNA. It is possible that we would detect small increases in NF-κB with higher doses of CpG.

IL-8 can also be regulated by post transcriptional modifications, therefore we investigated the role of CpG on mRNA stabilization. Addition of CpG to IL-1β-treated cells increased the half-life of IL-8 mRNA by 20 minutes. While a 20 minute increase in mRNA half-life may seem modest, it can affect mRNA abundance by orders of magnitute (for review [22]). A few other incidences of CpG increasing mRNA stability have been reported. CpG was found to increase LPS-induced TNFα mRNA stability in a murine macrophage-like cell line (RAW cells) [23]. Class 1 major histocompatability complex (MHC) mRNA levels were stabilized following treatment with CpG in dendritic cells [24]. In addition, there are many studies showing that activation of p38 stabilizes IL-8 mRNA [25,26]. We present evidence that CpG increased p38 phosphorylation, and pretreatment with SB202190, a chemical inhibitor of p38, abolished CpG-induced IL-8 expression. Collectively these data suggest the mechanism by which CpG increases IL-8 expression; upregulation of IL-8 mRNA transcription by IL-1β and enhanced IL-8 mRNA stability by CpG induction of p38. Together these events ultimately lead to increased IL-8 protein synthesis and secretion.

The mRNA of many inflammatory cytokines contain AU-rich elements (ARE) in their 3' untranslated regions which regulate its stability. IL-8 mRNA contains several AUUUA motifs in AU rich regions. The 3' tail is susceptible to deadenylation and degradation by endonucleases and/or 3'-5' exonucleases (reviewed in [27]). Mitogen activating protein kinase (MAPK) activating protein kinase-2 (MAPKAPK-2) is a downstream substrate of p38 and regulates a variety of proteins which regulate mRNA stabilization, including HuR and TTP (reviewed in [28]). HuR has been shown to bind AREs with high affinity and block the decay of deadenylated mRNA resulting in mRNA stabilization [28]. It is possible that p38 is regulating HuR activation, although we did not test that in this manuscript. TPP, on the other hand, acts to promote deadenylation to destabilize mRNA. It has been proposed that p38 phosphorylates TPP which causes it downregulation, resulting in mRNA stabilization [28]. In this study, we did not investigate which of the ARE binding proteins were being regulated by CpG-induced activation of p38. It is conceivable that both HuR and TPP could be activated and work in synergy to increase the stabilization of IL-8 mRNA.

TLR9 is expressed on a variety of airway epithelial cell types including 16HBE14o- (present study and [9]), tracheal and bronchial epithelium [9], respiratory epithelium from large airways resected during surgery [8], primary bronchial epithelial cells[10], and BEAS-2B cells [10]. CpG activation in human primary B cells and macrophages has been shown to be due to internalization of the DNA [12,29]. By selectively deleting TLR9 using siRNA, we showed the requirement of TLR9 in CpG-induced synergy. The use of chloroquine, an inhibitor of vesicular acidification, suggested that bacterial DNA is internalized in human airway epithelial cells; however this was not demonstrated in the present study.

In conclusion, we have shown that CpG modulates the expression of cytokine-derived IL-8 expression by increasing the phosphorylation of p38 leading to an increased half-life of IL-8 mRNA. Since NF-κB is crucial for transcriptional regulation of IL-8, a stimulus which increases NF-κB; i.e. IL-1β, TNFα, LPS, etc., is required for the initial increase in IL-8 transcription. CpG, through TLR9, contributes by stabilizing the existing mRNA. This study suggests an important role for CpG DNA in augmenting the immune response in human airway epithelium.

## Abbreviations

IL, interleukin; NF, nuclear factor; ODN, oligodeoxynucleotide; TLR, toll like receptor; TNF, tumor necrosis factor

## Competing interests

The authors (NWP, VSH, KML, HRW and KP) declare that they have no competing interests.

## Authors' contributions

NWP carried out the immunoassays and helped prepare the manuscript. VSH performed the Northern's and EMSA's. KML performed the Western blots and ELISA's. HRW conceived of the study and helped with manuscript preparation. KP designed and coordinated the study, and drafted the manuscript. All authors read and approved of the final manuscript.
